# A novel phase variant of the cholera pathogen shows stress-adaptive cryptic transcriptomic signatures

**DOI:** 10.1186/s12864-016-3233-x

**Published:** 2016-11-14

**Authors:** Bliss Lambert, Maheshi Dassanayake, Dong-Ha Oh, Shana B. Garrett, Sang-Yeol Lee, Gregg S. Pettis

**Affiliations:** 1 Department of Biological Sciences, Louisiana State University, Baton Rouge, Louisiana USA; 2 Division of Applied Life Science, Gyeongsang National University, Jinju, 660-701 South Korea

**Keywords:** Cholera, *Vibrio cholerae*, Phase variation, Transcriptomics, Bioinformatics, RNA sequencing

## Abstract

**Background:**

In a process known as phase variation, the marine bacterium and cholera pathogen *Vibrio cholerae* alternately expresses smooth or rugose colonial phenotypes, the latter being associated with advanced biofilm architecture and greater resistance to ecological stress. To define phase variation at the transcriptomic level in pandemic *V. cholerae* O1 El Tor strain N16961, we compared the RNA-seq-derived transcriptomes among the smooth parent N16961, its rugose derivative (N16961R) and a smooth form obtained directly from the rugose at high frequencies consistent with phase variation (N16961SD).

**Results:**

Differentially regulated genes which clustered into co-expression groups were identified for specific cellular functions, including acetate metabolism, gluconeogenesis, and anaerobic respiration, suggesting an important link between these processes and biofilm formation in this species. Principal component analysis separated the transcriptome of N16961SD from the other phase variants. Although N16961SD was defective in biofilm formation, transcription of its biofilm-related *vps* and *rbm* gene clusters was nevertheless elevated as judged by both RNA-seq and RT-qPCR analyses. This transcriptome signature was shared with N16961R, as were others involving two-component signal transduction, chemotaxis, and c-di-GMP synthesis functions.

**Conclusions:**

Precise turnarounds in gene expression did not accompany reversible phase transitions (i.e., smooth to rugose to smooth) in the cholera pathogen. Transcriptomic signatures consisting of up-regulated genes involved in biofilm formation, environmental sensing and persistence, chemotaxis, and signal transduction, which were shared by N16961R and N16961SD variants, may implicate a stress adaptation in the pathogen that facilitates transition of the N16961SD smooth form back to rugosity should environmental conditions dictate.

**Electronic supplementary material:**

The online version of this article (doi:10.1186/s12864-016-3233-x) contains supplementary material, which is available to authorized users.

## Background

*Vibrio cholerae* is a Gram-negative rod-shaped bacterium that is naturally ubiquitous in coastal, estuarine, and riverine waters in planktonic form and within biofilms associated with abiotic and biological materials [1]. Toxin-producing strains of *V. cholerae* cause the serious diarrheal disease cholera. Epidemic strains undergo a reversible phase variation event between smooth and rugose colonial morphotypes at a frequency apparently greater than that of non-epidemic clinical and environmental strains [2]. Rugose cells form highly corrugated colonies and more structurally complex biofilms due to excess production of *Vibrio* polysaccharide (VPS). VPS is a viscous biopolymer partly composed of structural matrix proteins and a polysaccharide (VPS-PS) containing glucose, galactose and an amide between 2-acetamido-2-deoxy-α-guluronic acid and glycine [3–5]. The biofilm proficient rugose phase facilitates nutrient acquisition from insubstantial sources such as drinking water reservoirs, enhances resistance to chlorine and a variety of environmental stresses [6–8] and provides greater resistance to complement-mediated serum lysis [9, 10]. Consequently, rugosity is considered a survival adaptation that enhances the overall fitness of *V. cholerae* in aquatic habitats and may additionally contribute to the pathogenesis of the organism [11, 12].

The genes responsible for assembly and transport of VPS-PS are distributed across two closely positioned loci, *vpsI* and *vpsII*, which are situated within an approximately 30.7 kb region on the larger of the two chromosomes of *V. cholerae* [4]. Most of the *vps* gene products play various biosynthetic and functional roles in the formation of VPS-PS, while several others are hypothetical proteins of unknown function [13, 14]. Characterization of non-polar *vps* deletion mutants revealed that most of the *vps* genes are required for the production of corrugated colonies, pellicles, and biofilms [14]. The two *vps* gene clusters are separated by an intergenic region that contains the *rbm* genes, which encode biofilm matrix proteins unique to *V. cholerae* [15]. Bap1, encoded by the unlinked VC1888 gene, is a homolog of one of the Rbm proteins and is also required for *V. cholerae* biofilm stability [14, 15].

Regulation of VPS production in *V. cholerae* is quite complex. It is positively controlled by the transcriptional regulators VpsR and VpsT, which are both required for corrugated colony and biofilm formation [16, 17]. VpsR is a stronger regulator of the *vps* genes than is VpsT and acts together with the alternative sigma factor RpoN. VPS production and biofilm formation are favored by increasing concentrations of the second messenger molecule, c-di-GMP [18, 19], which is synthesized by GGDEF-domain diguanylate cyclases (DGCs) and degraded by EAL- or HD-GYP-domain phosphodiesterases (PDEs). The expression of *vps* genes is negatively regulated by quorum sensing through the master regulator HapR [7, 20–22]. Some strains of *V. cholerae*, including pandemic O1 El Tor strain N16961, contain natural nonsense mutations in their *hapR* genes [13, 23]. While it was once thought that *hapR* mutant strains were unable to regulate gene expression in response to changes in bacterial populations, more recent studies have demonstrated that even in the absence of a functional *hapR* gene, other quorum sensing components are able to circumvent the normal HapR-dependent pathway to regulate gene expression [24].

A microarray analysis completed by Yildiz et al. [21] identified 124 differentially regulated genes between smooth and rugose phase variants of the *V. cholerae* O1 El Tor A1552 strain. Biofilm-related genes, including *vps* and *rbm*, as well as genes coding for activated sugar nucleotide intermediates, secreted proteins, and putative chitinases, were found to be up-regulated in rugose phase variants. Among the genes that were down-regulated in rugose as compared to smooth were flagellar motility and several chemotaxis-related genes.

RNA-seq technology has recently been used to detect genome-wide transcriptional regulation in *V. cholerae* and has also been used in combination with ChIP-seq to effectively resolve certain regulons [25–28]. Here we used RNA-seq to obtain a comprehensive overview of the whole genome expression changes that occur between smooth and rugose colonial phase variants of *V. cholerae* N16961. Because phase variation can be a reversible process, we included a set of smooth phase variants that were directly derived from rugose isolates in addition to the original smooth parental variants in our analysis. Our results implicate specific metabolic changes, including production and utilization of acetate and anaerobic respiration, which were not previously linked to colonial phase transitions in *V. cholerae*. Phenotypic and transcriptomic characterization of the smooth variants derived from rugose revealed them to be distinct from the original smooth parent in that they were deficient in biofilm formation despite having *vps* and *rbm* transcripts at elevated levels reminiscent of the rugose isolates. Moreover, we found similar shared transcriptomic signatures between the rugose and their smooth derivatives for genes related to acetate and peptide metabolism, as well as some that encode for regulatory functions and chemotaxis proteins.

## Results and discussion

### Isolation and phenotypic characterization of colonial phase variants

**Fig. 1:**
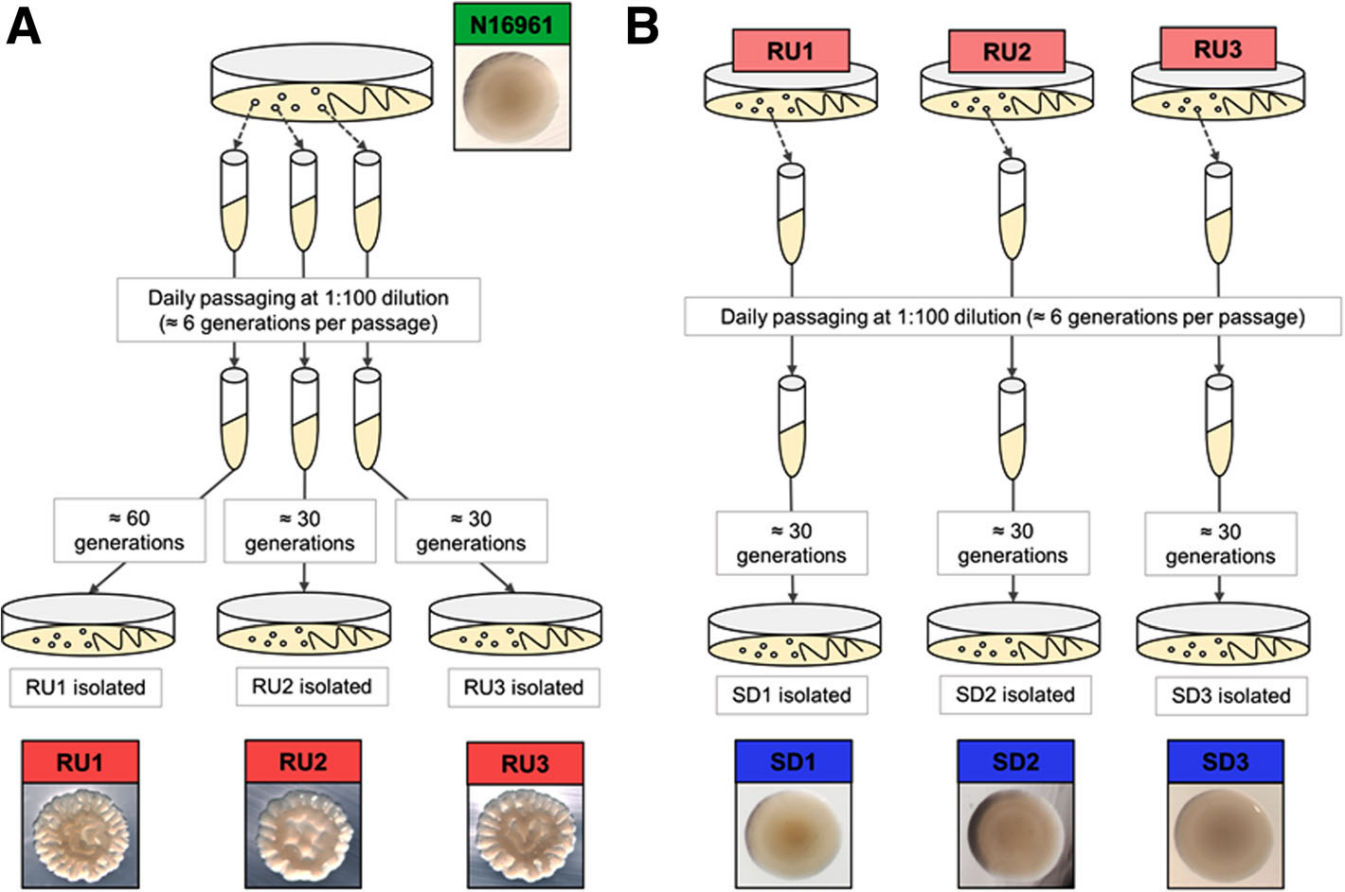
Experimental procedure for isolation of N16961-derived phase variants. **a** Beginning with isolated colonies, parental strain N16961 was subjected to daily passaging with plating following the indicated approximate number of bacterial generations. Single randomly selected rugose isolates, designated RU1, RU2 and RU3, were then chosen. In the passaging experiment where RU1 was eventually selected, plating was also performed after ~30 generations, but there were no clearly distinguishable rugose isolates present on those plates; **b** Beginning with isolated colonies of RU1, RU2, and RU3, the passaging and plating procedure was repeated, and single randomly chosen smooth isolates, designated SD1, SD2 and SD3, were selected at the end

Starting with three well-isolated smooth colonies of the parental strain N16961, independent broth cultures were passaged daily with occasional plating for individual colonies. Rugose (N16961R) variants were eventually identified, and a single representative was randomly chosen from each passaging experiment for further study (i.e., RU1, RU2, RU3 in Fig. 1a). Similar daily passaging and occasional plating beginning with strains RU1, RU2, and RU3 eventually yielded smooth derivative (N16961SD) isolates, and single randomly chosen representatives were again selected (i.e., as shown in Fig. 1b, isolates SD1, SD2, and SD3 were selected from assays beginning with RU1, RU2, and RU3, respectively). Multiple independent quantitative switching assays (see Methods for details) beginning with each of the N16961R variants yielded smooth colonies at frequencies ranging from 1.7 ± 0.2% to as much as 58.9 ± 4.9% of the total colonies counted and phenotypically scored for a given assay. Such high frequency switching was similar to that observed for the initial conversion of N16961 to N16961R (data not shown) and thus was consistent with a reversible phase variation event(s). Previously, Yildiz and Schoolnik [4] reported reversible switching between smooth and rugose forms of *V. cholerae* strain A1552 at frequencies comparable to ours.

**Fig. 2:**
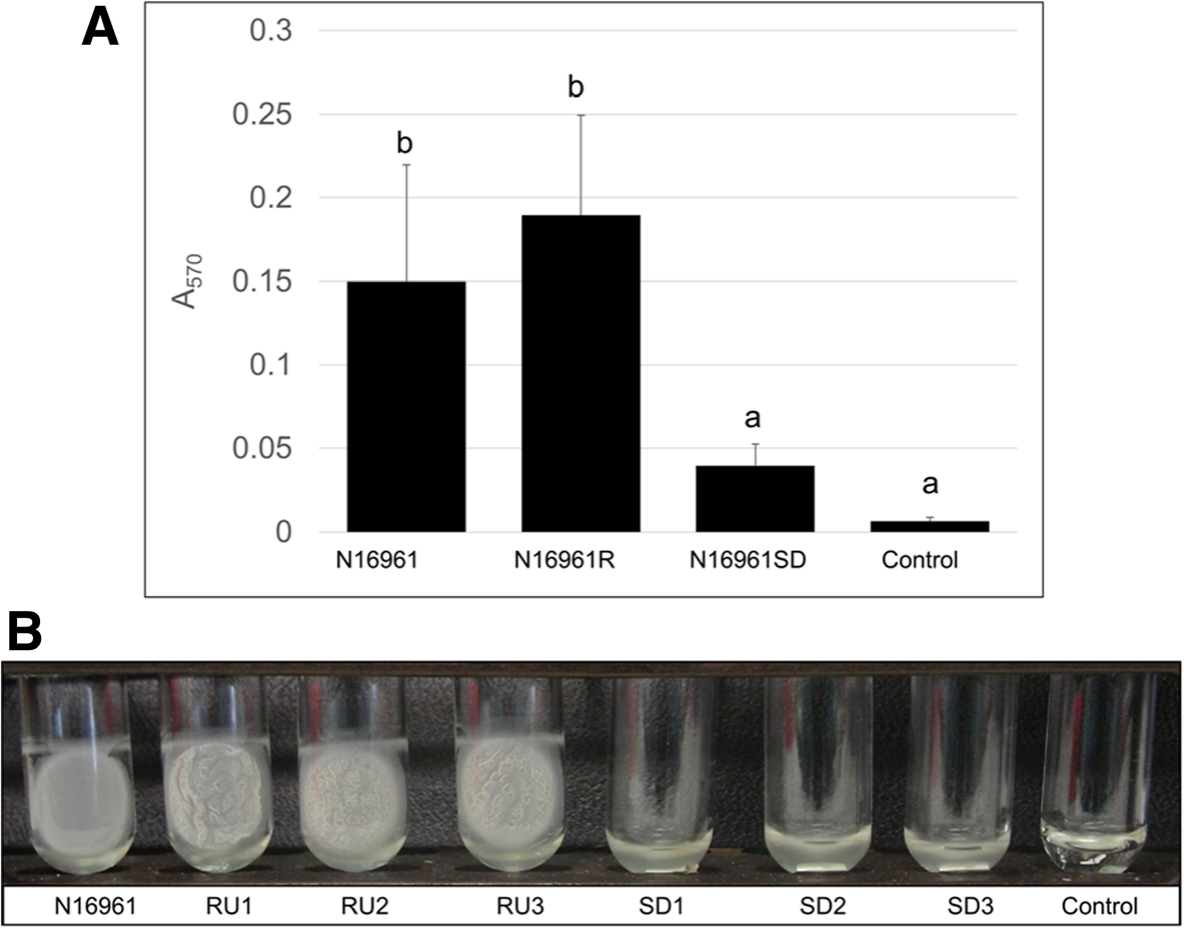
Biofilm formation of N16961 phase variants. **a** Following 48 h of static incubation at 30 °C, liquid cultures were poured off and the remaining attached biofilm material was stained with 0.1% crystal violet and quantified by measuring the absorbance at 570 nm. Represented in the graph are the average absorbance values of 12 replicates of N16961, 18 replicates (6 per individual variant) of N16961R, 18 replicates (6 per individual variant) of N16961SD, and 6 replicates of the uninoculated control. Error bars show standard deviations. Samples indicated with the same letter were not significantly different according to Tukey’s post-test (*P* < 0.05). **b** Following 48 h of static incubation at 30 °C, liquid cultures were carefully poured out and the pellicles that had formed at the air-broth interface during incubation were retained and repositioned at the side of each glass tube to be photographed

**Fig. 3:**
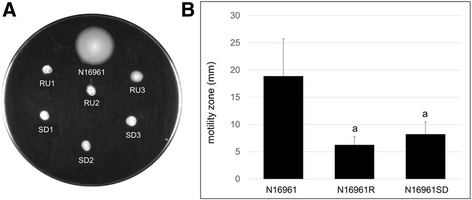
Motility of N16961 phase variants. **a** Representative plate showing motility zones of variants following overnight incubation. **b** Following overnight incubation of motility plates, motility zones were measured in mm. Represented in the figure are the averages of 10 plates, with error bars depicting the standard deviations. Samples indicated with same letter were not statistically different according to Tukey’s post-test (*P* < 0.05)

Growth curves of N16961, N16961R and N16961SD isolates revealed that N16961 and N16961SD strains all had doubling times of approximately 25 min during exponential phase growth, while N16961R strains grew somewhat slower with a doubling time of approximately 30 min (Additional file 1: Figure S1). We also examined phase variants for biofilm formation and motility, two attributes that have been used previously to distinguish smooth and rugose forms of *V. cholerae*. In biofilm tube assays, the quantity of biofilm material produced by N16961R variants was trending towards a significant difference from that produced by the N16961 parent according to statistical analysis with Tukey’s post-test (*P* = 0.06) (Fig. 2a). Inspection of pellicles produced by N16961 and N16961R samples revealed differences in biofilm architecture (Fig. 2b) in that pellicles formed by N16961 appeared smooth at the surface and were easily disrupted with agitation, while pellicles formed by all three of the N16961R variants appeared wrinkled and were not easily disrupted by vortexing. Meanwhile, N16961SD variants did not produce obvious biofilms (Fig. 2a and b) and, in fact, samples within the N16961SD group were not statistically different from the uninoculated controls (*P* = 0.37). In motility assays, both N16961R and N16961SD variants were significantly less motile than N16961 (*P* = 1.01E-05) (Fig. 3).

### Principal component analysis of RNA-seq data

Total RNA was isolated from nine mid-exponential cultures of N16961, N16961R and N16961SD phase variants (i.e., from three independent cultures of N16961, and one culture each of RU1, RU2, RU3, SD1, SD2 and SD3). The RNA was depleted of DNA and rRNA and constructed into strand-specific barcoded cDNA libraries, which were then sequenced on a single flowcell of an Illumina HiSeq2000 to give 196,433,469 total reads, each of 100 nucleotides in length. Individual samples ranged from approximately 18 to 24 million reads. The short reads generated for this project were deposited at the NCBI SRA database under accession number PRJNA295073. For each sample, greater than 99% of the high quality reads were mapped to the reference *V. cholerae* N16961 genome reported by Heidelberg et al. [13] (Additional file 2: Table S1). Reads that did not exclusively map well within the confines of individual predicted gene models were excluded from further analysis. The remaining read counts normalized in fragments per kilobase per million reads (FPKM) for each sample with regard to gene models identified in the reference genome are given in Additional file 3: Table S2.

**Fig. 4:**
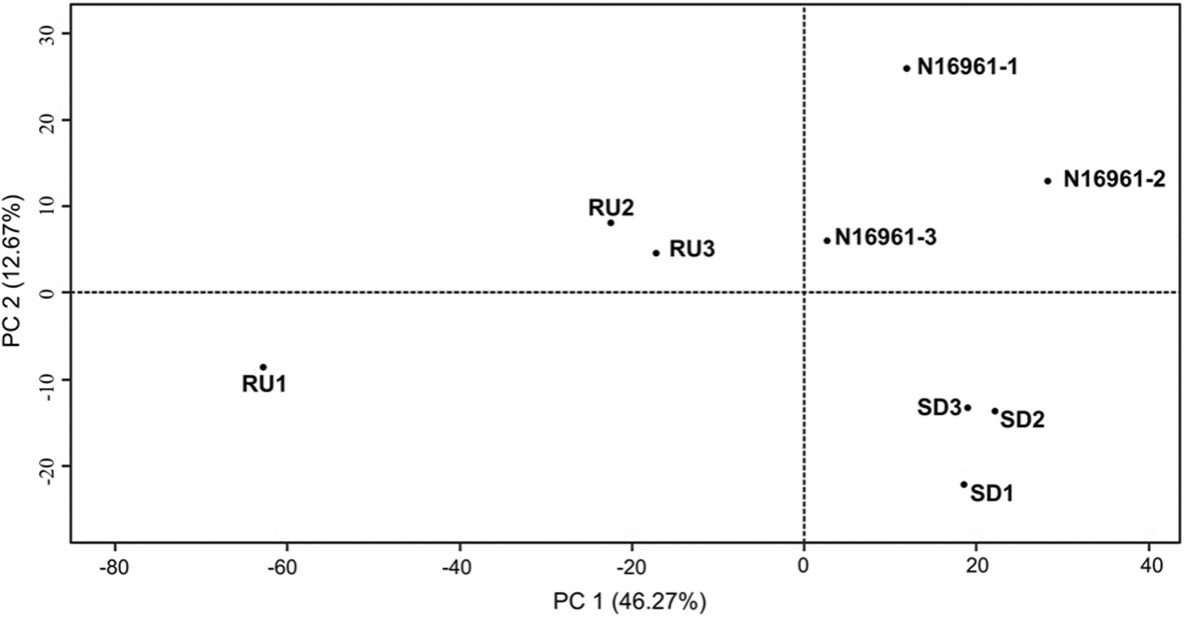
Principal Component Analysis. The principal components (PC 1 and PC 2) identified together account for 58.94% of the overall variability of the dataset. Phase variants were grouped by PCA based on transcriptomic similarities

These reads were then clustered in a principal component analysis (PCA), which confirmed the three N16961, N16961R or N16961SD transcriptomes were more similar to one another than they were to those of the other phase variant groups (Fig. 4, Additional file 4: Figure S2; Additional file 5: Figures S3; Additional file 6: Figure S4). The RU1 sample was separated by PCA from the other two samples within the N16961R phase variant group. Although RU1 was distinct from the other two N16961R isolates, the expression profile of SD1, the smooth derivative that was isolated from RU1, was very similar to the expression profiles of the other two N16961SD isolates. Indeed, the N16961SD samples were the most clustered grouping in the PCA analysis, sharing even more transcriptomic similarities than the N16961 parental group. Principal component 1 (PC1) separated the parental and the N16961SD group, both of which share the smooth colonial phenotype, from the rugose variants. The parental group and N16961SD group, however, were different in motility and capacity to form biofilms (Figs. 2 and 3), and their transcriptomes were separated based on principal component 2 (PC2). This result also implies that the N16961SD phase variants did not arise from an exact reversal of the adjustments in gene expression that occurred concomitantly with the initial smooth-to-rugose switch, a conclusion that is supported by subsequent analysis of the RNA-seq data as detailed below.

### Patterns and functional categories of differential gene expression identified by RNA-seq analysis

Differential expression analysis of the normalized RNA-seq data was performed using DESeq [29], which identifies differentially expressed genes using a negative binomial distribution model and corrects for false discovery rate at 5% by generating *P*_adj_ values using the Benjamini and Hochberg method [30]. Results of the three pairwise comparisons, i.e., the parents (N16961) versus the rugose variants (N16961R), N16961R versus the smooth derivatives (N16961SD), and N16961 versus N16961SD, are given in Additional file 7: Table S3; Additional file 8: Table S4; Additional file 9: Table S5. There were 62 genes significantly differentially expressed (*P*_adj_ < 0.05) between N16961 and N16961R, with 50 of them being up-regulated and 12 being down-regulated. The majority of these genes were not previously described by Yildiz et al. [21], which may be the result of multiple factors, including the increased resolution of RNA-seq compared to microarray analysis and the different strains examined. In comparison to our observed expression changes between N16961 and N16961R, more significant differences were observed here between N16961R and N16961SD phases, with 180 genes differentially expressed, of which 80 were up-regulated and 100 were down-regulated. The majority of significantly differentially expressed genes were clustered into co-expression groups, and functions represented in each group were identified based on the sequenced *V. cholerae* N16961 genome annotation [13]. The five gene expression patterns observed were: i) up-regulated in the transition from N16961 to N16961R and remaining up-regulated in the transition from N16961R to N16961SD (Additional file 10: Table S6); ii) up-regulated from N16961 to N16961R and down-regulated in N16961R to N16961SD (Additional file 11: Table S7); iii) down-regulated from N16961 to N16961R and up-regulated in N16961R to N16961SD (Additional file 12: Table S8); iv) not significantly regulated from N16961 to N16961R and then down-regulated in N16961SD (Additional file 13: Table S9); v) not significantly regulated from N16961 to N16961R and then up-regulated in N16961SD (Additional file 14: Table S10). For the differentially regulated genes identified here, we will focus our results and discussion on functional categories of genes that were also previously described by Yildiz et al. [21], as well as those newly described functions that we postulate may contribute either positively or negatively to biofilm development and associated phenotypic changes (e.g., rugosity).

### Sugar transport and utilization

Genes encoding components of the phosphoenolpyruvate phosphotransferase system (PTS), including VCA0653 (*srcA*), which encodes a sucrose-specific PTS component and VC1826, which encodes a putative fructose-specific PTS component, were both down-regulated at least 30 fold on the N16961 to N16961R switch and then up-regulated in the N16961R to N16961SD switch (Additional file 12: Table S8). Yildiz et al. [21] also observed a reduced expression of fructose-specific PTS components in their rugose isolate. The PTS is a phosphotransfer cascade that transports and phosphorylates specific carbohydrates, such as glucose, sucrose, fructose, mannose, and N-acetylglucosamine, into the cell [31]. The phosphorylation state of certain PTS components acts as a signal of environmental carbohydrate availability and these reversible phosphorylation signals influence the activation or inactivation of other cellular processes including biofilm formation [32, 33]. Our findings here appear to be consistent with previous results where fructose and sucrose were found to inhibit the formation of rugose colonies of *V. cholerae* [34]. Similar regulation of certain PTS sugar utilization genes was also observed here, including VCA0655, which encodes a sucrose-6-phosphate hydrolase that is required for utilization of sucrose as a sole carbon source, and VC1827 (*manA*), which is involved in mannose catabolism (Additional file 12: Table S8).

### VPS production and biofilm formation

**Fig. 5:**
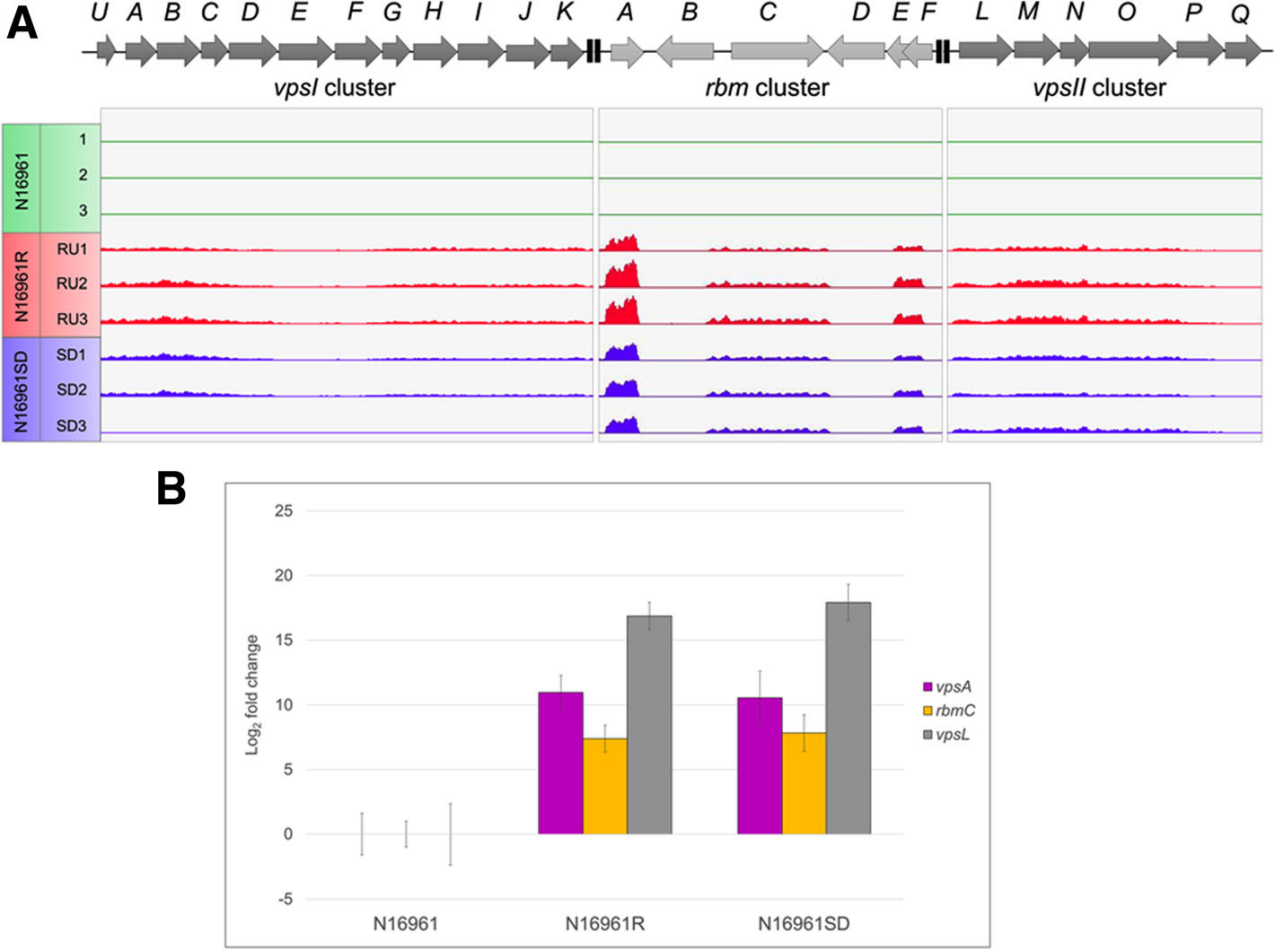
Transcription analysis of *vps* and *rbm* genes among N16961 phase variants. **a** Normalized RNA expression profiles based on RNA-seq results for biofilm-related genes. Peaks corresponding to the number of transcripts that mapped to regions containing the *vpsI*, *vpsII*, and *rbm* gene clusters are shown in parallel tracks for each phase variant. N16961 samples are depicted in green, N16961R samples are shown in red, and N16961SD samples are shown in blue. Although our analysis reported similar up-regulated expression values of the genes of all three clusters in the N16961R and N16961SD samples as compared to N16961, some genes of the *vpsI* cluster fell out of the range of statistical significance in the N16961SD samples (Additional file 10: Table S6). These include the *vpsI* gene, which encodes a glycosyltransferase, the *vpsE* and *vpsH* genes, whose products are predicted to be involved VPS export, and the *vpsF* and *vpsJ* genes, which encode proteins of unknown function. The increased *P*_*adj*_ values obtained for these genes may be a result of the apparent differences in expression of the *vpsI* cluster in the SD3 sample versus SD1 and SD2, which can be seen in the figure. **b** RT-qPCR verification of up-regulation of genes representative of the *vpsI*, *vpsII*, and *rbm* gene clusters in N16961R and N16961SD samples. The graph depicts the log_2_ fold changes of gene transcripts calculated for each selected gene relative to their transcript abundances in the N16961 sample group at a confidence interval of 95%. All data were normalized respective to the reference gene, *gyrA*, and the error bars depict the normalized quantity standard error

Fifteen of the 24 *vps* and *rbm* genes were significantly up-regulated following the transition from N16961 to N16961R with fold changes ranging from approximately 12 to nearly 200 (Additional file 10: Table S6). These genes were not significantly down-regulated from N16961R to N16961SD, and 11 of the 15 genes remained significantly up-regulated in N16961SD when compared with the N16961 transcriptome (Additional file 10: Table S6). In contrast to Yildiz et al. [21], we did not detect differential regulation of the *vpsU*, *vpsC*, *vpsG*, *vpsK*, *rbmE*, *vpsN*, *vpsP,* and *vpsQ* genes, while, neither study showed evidence of differential expression of the *rbmF* gene. Normalized RNA expression profiles of the *vpsI*, *vpsII* and *rbm* clusters for each of the phase variants were visualized (Fig. 5a) in parallel tracks using Integrative Genomics Viewer [35]. RNA peaks showed very similar qualitative expression patterns for all three clusters in the N16961R and N16961SD samples further supporting the consistent expression patterns observed within biological replicates.

To confirm these RNA-seq results, particularly the unexpected up-regulation of a number of *vps* and *rbm* genes in the biofilm-defective N16961SD variants, we also performed RT-qPCR on representative differentially regulated genes of the *vpsI, vpsII* and *rbm* clusters. Consistent with the RNA-seq analysis, significantly higher levels of the *vpsA*, *vpsL* and *rbmC* transcripts were observed in the N16961R and N16961SD samples relative to N16961 (Fig. 5b). Furthermore, there was consistency between the RNA-seq and RT-qPCR analyses regarding the order of magnitude of induction (with *vpsL* being the most up-regulated, followed by *vpsA*, and then *rbmC*) and with the fact that for each method a given gene’s induction levels in N16961R and N16961SD relative to N16961 were similar. RT-qPCR was performed initially by using aliquots of RNA from the original samples used for RNA-seq. When the RT-qPCR was repeated using freshly prepared independent RNA from the nine isolates, nearly identical results were obtained (data not shown).

The *vps* and *rbm* genes are known to be positively regulated at the transcriptional level by the action of VpsR and the alternative sigma factor RpoN [16]. Indeed, VC0665, which encodes the RpoN-dependent VpsR protein, was up-regulated greater than 10-fold from N16961 to N16961R and remained up-regulated in N16961SD isolates (Additional file 10: Table S6). Another transcriptional regulator, CdgA, which is positively regulated by VpsR and functions as a diguanylate cyclase to increase c-di-GMP levels and increase transcription of the *vps* and *rbm* genes [36], was also up-regulated greater than 5-fold from N16961 to N16961R and its expression then did not significantly change upon transition to N16961SD (Additional file 10: Table S6).

The VC1888 gene encoding a homologue of RbmC, Bap1, which is required for biofilm integrity [15], was also found to be up-regulated in the N16961 to N16961R switch and remained up-regulated in the N16961SD samples (Additional file 10: Table S6). The elevated *vps* and *rbm* transcript levels in the N16961SD isolates raise the possibility that post-transcriptional, or perhaps post-translational, regulation of one or more of these genes or their gene products has occurred, and that such regulation may be the basis for the biofilm-defective phenotype of this phase variant. Interestingly, negative regulation of *rbmC* transcript translation was recently shown to result from binding of a sRNA [37].

### Acetate production and utilization and central metabolism

The VC0298 (*acs*) gene, which encodes acetyl-coenzyme A synthetase (ACS), was found to be up-regulated approximately 4-fold upon the switch from N16961 to N16961R (Additional file 10: Table S6). The ACS protein is part of a high affinity bacterial pathway used to scavenge (assimilate) acetate from the environment. In *E. coli*, such assimilation has been shown to occur just as cells transition to a slower growth phase (e.g., stationary phase). Prior to this event, acetate is excreted (dissimilated) during exponential growth; thus, this represents a change in acetate production and usage with induction of *acs* transcription being part of the mechanism that flips the switch [38]. The implication from the RNA-seq data for VC0298 is that a switch to acetate assimilation is associated with rugosity in *V. cholerae*, at least under the growth conditions used in our study.

While ACS catalyzes the formation of acetyl-CoA from acetate during assimilation, the reverse pathway (i.e., acetyl-CoA conversion to acetate through the intermediate acetyl-P) is achieved during dissimilation by the sequential action of phosphate acetyltransferase (PTA) and acetate kinase (ACKA) enzymes, which in *V. cholerae* are encoded by the VC1097 and VC1098 genes, respectively. Interestingly, while transcription of the *acs* gene was not significantly different between N16961R and N16961SD isolates (Additional file 10: Table S6), transcript abundance of both the VC1097 and VC1098 genes was increased by nearly 3-fold in the smooth derivative compared to rugose (Additional file 14: Table S10). Moreover, two genes, VC2413 (*aceF*) and VC2414 (*aceE*), encoding components of the pyruvate dehydrogenase complex that converts pyruvate to acetyl-CoA, were also up-regulated approximately 3-fold in the N16961SD variants (Additional file 14: Table S10). These data raise the possibility that a switch back to acetate production and excretion is associated with the smooth derivative.

As shown in *E. coli*, utilization of acetate for growth necessarily involves the glyoxylate bypass (GB) and gluconeogenesis [38]. The expression of the malate synthase gene VC0734 (*aceB*), whose product is a critical enzyme controlling metabolic flux specifically through the GB, was induced approximately 6-fold from N16961 to N16961R and then down-regulated nearly the same amount in N16961SD (Additional file 11: Table S7). Also, the gene for 2-methylcitrate synthase, VC1337 (*prpC*), which functions in both the TCA cycle and GB, was similarly regulated (Additional file 11: Table S7). Other TCA/GB genes, including VC2092 (*gltA*), VC1338 (*acnA*), VC0604 (*acnB*) and VC1141 (*icd*), or TCA only genes VC2086 (*sucB*) and VC2085 (*sucC*), were not significantly changed from N16961 to N16961R but then were all reduced by 3-fold or greater in N16961SD (Additional file 13: Table S9). Meanwhile, genes involved in gluconeogenesis, including VCA0987 (*ppsA*), VC2738 (*pck*), and VC2544 (*fbp*), were not significantly regulated between N16961 and N16961R but were then all down-regulated 2-fold or greater in N16961SD (Additional file 13: Table S9). Overall, our data provide evidence of induction of GB in N16961R with reduction of this pathway (and TCA), along with gluconeogenesis, in N16961SD. It is tempting to speculate that for the rugose isolates here the combination of acetate assimilation, GB, and gluconeogenesis resulted in conversion of acetate to glucose, which is a component of VPS-PS [5]; thus, these metabolic pathways may play significant roles in biofilm formation in this species. Interestingly, besides its potential role in biofilm formation, acetate assimilation in *V. cholerae* also appears to affect the cholera disease process itself by altering host insulin signaling and metabolism of lipids [39].

### Anaerobic respiration and growth

Genes involved in anaerobic respiration including VC1514 and VC1511, which code for proteins with putative formate dehydrogenase activity, and VC1516, which encodes an iron-sulfur protein, were up-regulated approximately 4-5-fold in the switch from N16961 to N16961R, and this up-regulation was reversed upon the switch from N16961R to N16961SD (Additional file 11: Table S7). Other predicted anaerobic respiration or fermentation genes were not significantly regulated from N16961 to N16961R but then were down-regulated in N16961SD derivatives. These included VCA0678 (*napA*), which encodes a periplasmic nitrate reductase, VC2656 (*frdA*), encoding fumarate reductase, and VCA0984 (*lldD*), encoding lactate dehydrogenase (Additional file 13: Table S9). In the case of the latter function, it is possible that down-regulation of lactate production in N16961SD allows for more pyruvate to be available for putative acetate fermentation. Other genes whose products are predicted to function under anaerobic conditions were also down-regulated in N16961SD variants, including VCA0511 (*nrdD*), encoding a ribonucleoside triphosphate, VCA0665 (*dcuC*), encoding a C4-dicarboxylate transporter, VC0667 (*tas*), encoding an aldo/keto reductase, and VC1950 (*torZ*), encoding a trimethylamine-N-oxide reductase (Additional file 13: Table S9). The results for genes in this category implicate a role for oxygen limitation/anaerobiosis in rugosity and potentially biofilm formation in *V. cholerae*. A role for oxygen deprivation in biofilm development in this organism was also postulated based on proteomic studies performed for cells grown under differing oxygen conditions [40].

### Motility and chemotaxis

In contrast to our current findings, Yildiz et al. [21] reported that expression of some class III and IV flagellar genes was reduced in the strain A1552 rugose variant, whose motility was shown to be reduced by about 50%; however, the significance of this finding was unclear since swimming behavior and flagella production seemed unaffected compared to the A1552 smooth parent. We found that while motility of N16961R was significantly reduced relative to N16961 by over 3-fold (Fig. 3), there was no concomitant down-regulation of flagellar genes. We did observe down-regulation of some class I and class II flagellar genes ranging from fold changes of approximately 2–4 in the switch from N16961R to N16961SD (Additional file 13: Table S9). The significance of this regulation is similarly unclear since motility appeared to be unchanged between these two types of variants.

The previous microarray analysis also identified several differentially regulated chemotaxis genes [21]. One of those identified previously as being up-regulated in rugose, VCA0864, was also up-regulated over 19-fold in N16961R and remained up-regulated in N16961SD (Additional file 10: Table S6) in our study. The gene for another methyl-accepting chemotaxis protein (VC1859) was also up-regulated by nearly 6-fold in N16961R but was then down-regulated by the same amount in N16961SD (Additional file 11: Table S7). Additionally, some genes encoding chemotaxis-related functions were unchanged in the initial transition but then were down-regulated in the N16961SD samples, including the methyl accepting chemotaxis proteins encoded by the VC1298, VC1413, VC2161, VCA0658, and VCA0773 genes (Additional file 13: Table S9).

### Additional regulatory functions

The putative c-di-GMP synthetase gene, VC2224, was up-regulated over 6-fold in N16961R and then down-regulated 3.4-fold in N16961SD (Additional file 11: Table S7). Expression of a putative c-di-GMP synthetase with an extracellular solute binding domain encoded by the VCA0557 gene was unchanged in N16961R compared to N16961 but was then decreased by nearly 3-fold in N16961SD (Additional file 13: Table S9). The VCA0785 gene, which also encodes a protein with predicted c-di-GMP synthetase and phosphodiesterase activity was found to be up-regulated over 10-fold in both the N16961R and N16961SD variants as compared to N16961 (Additional file 10: Table S6). As corrugated colony formation, VPS synthesis, and biofilm formation have all been previously reported to be controlled by intracellular c-di-GMP levels in *V. cholerae*, the differentially regulated genes related to c-di-GMP synthesis identified in our analysis are likely to be involved in mediating phenotypic changes that occur with colonial phase variation in this organism.

The gene VC1349, which encodes a PAS domain-containing sensor histidine kinase protein of a bacterial two component system, was up-regulated in N16961R (in agreement with Yildiz et al. [21]), and it remained up-regulated in N16961SD (Additional file 10: Table S6). Similarly, the VC1348 gene, a putative response regulator cognate to the VC1349 product was induced in N16961R and remained highly expressed in N16961SD (Additional file 10: Table S6). The presence of a HD-GYP domain in this response regulator suggests that it probably possesses PDE activity, which would act to degrade c-di-GMP in response to particular environmental stimuli.

Other genes encoding signal sensing proteins that were found to be down-regulated in N16961SD were VC1085, VC1315, and VC1710, encoding sensor kinase proteins, and the VC1081 gene, which might be the cognate response regulator for VC1085 (Additional file 13: Table S9). The VC1710 gene contains both an EAL phosphodiesterase domain and a CBS domain, with the latter being associated with adenosyl (AMP, ATP, or SAM) binding.

### Peptide transport and utilization

The VC0194 (*ggt*) gene encoding gamma-glutamyl transpeptidase (GGT), which was up-regulated in *V. cholerae* strain A1552 rugose previously [21], was found here to be 15-fold higher in N16961R than N16961 and was then reduced approximately 4-fold from N16961R to N16961SD (Additional file 11: Table S7). GGT is required for the utilization of exogenous gamma-glutamyl peptides and facilitates de novo synthesis of cysteine and glycine, which is a component of VPS-PS [5]; thus, it is possible that GGT contributes significantly to VPS-PS production in rugose *V. cholerae*. Interestingly, GGT was reported to contribute to the environmental persistence of *E. coli* under growth-limiting conditions [41, 42], and it has also been implicated as a colonization factor of the bacterial pathogens *Helicobacter pylori* and *Campylobacter jejuni* [43, 44].

Two oligopeptide permease components encoded by VC1091 (*oppA*) and VC1092 (*oppB*) were up-regulated about 5-fold in N16961R and were then down-regulated over 8-fold in the N16961SD variants (Additional file 11: Table S7). In addition to the role of peptide transport systems in cell nutrition, they have also been implicated in other processes such as chemotaxis, conjugation, virulence, and competence [45]. Additionally, an *oppA* mutant of *Vibrio fluvialis* was found to have increased biofilm formation when grown in media containing peptone or tryptone as a nitrogen source [46].

### Stress response

Some genes involved in stress response were up-regulated in the N16961SD derivatives (Additional file 14: Table S10). These genes include VC0855 (*dnaK*) and VC0856 (*dnaJ*), which were both up-regulated approximately 3-fold, and, interestingly, were found to be differentially regulated in *hapR* mutants in the microarray study by Yildiz et al. [21]. The protein products of these two genes act as chaperones to protect other proteins from damage during stressful conditions such as heat shock. Additionally, the VCA0183 (*hmp*) gene was up-regulated more than 2.4-fold. Hmp is a nitric oxide dioxygenase, which uses O_2_ and NADPH to convert nitric oxide into nitrate, conferring greater resistance to nitrosative stress. Finally, the VC2506 (*rapA*) gene was up-regulated 2.5-fold. The *rapA* gene encodes an RNA polymerase associated protein, which stimulates the recycling of RNA polymerase during transcription in stressful conditions.

## Conclusion

Although phase variation in *V. cholerae* between smooth and rugose forms was known to be phenotypically reversible, smooth variants derived from rugose had not previously been analyzed in any detail. The N16961SD isolates are biofilm-deficient apparently due to an uncharacterized genetic or epigenetic change which still allows for elevated transcription of *vps* and *rbm* genes. Given the differential regulation of multiple genes involved in acetate metabolism in the smooth derivatives, an intriguing epigenetic possibility which would link underlying metabolic changes with biofilm forming capability would involve post-translational inactivation via acetylation (by either acetyl-CoA or acetyl phosphate) of one or more components required for rugosity. Regardless of the exact mechanism, however, it is apparent that although these smooth variants phenotypically resemble the N16961 parent in colony morphology, they nevertheless share certain cryptic transcriptomic signatures with the rugose isolates, at least under the culture conditions used in our study.

**Fig. 6:**
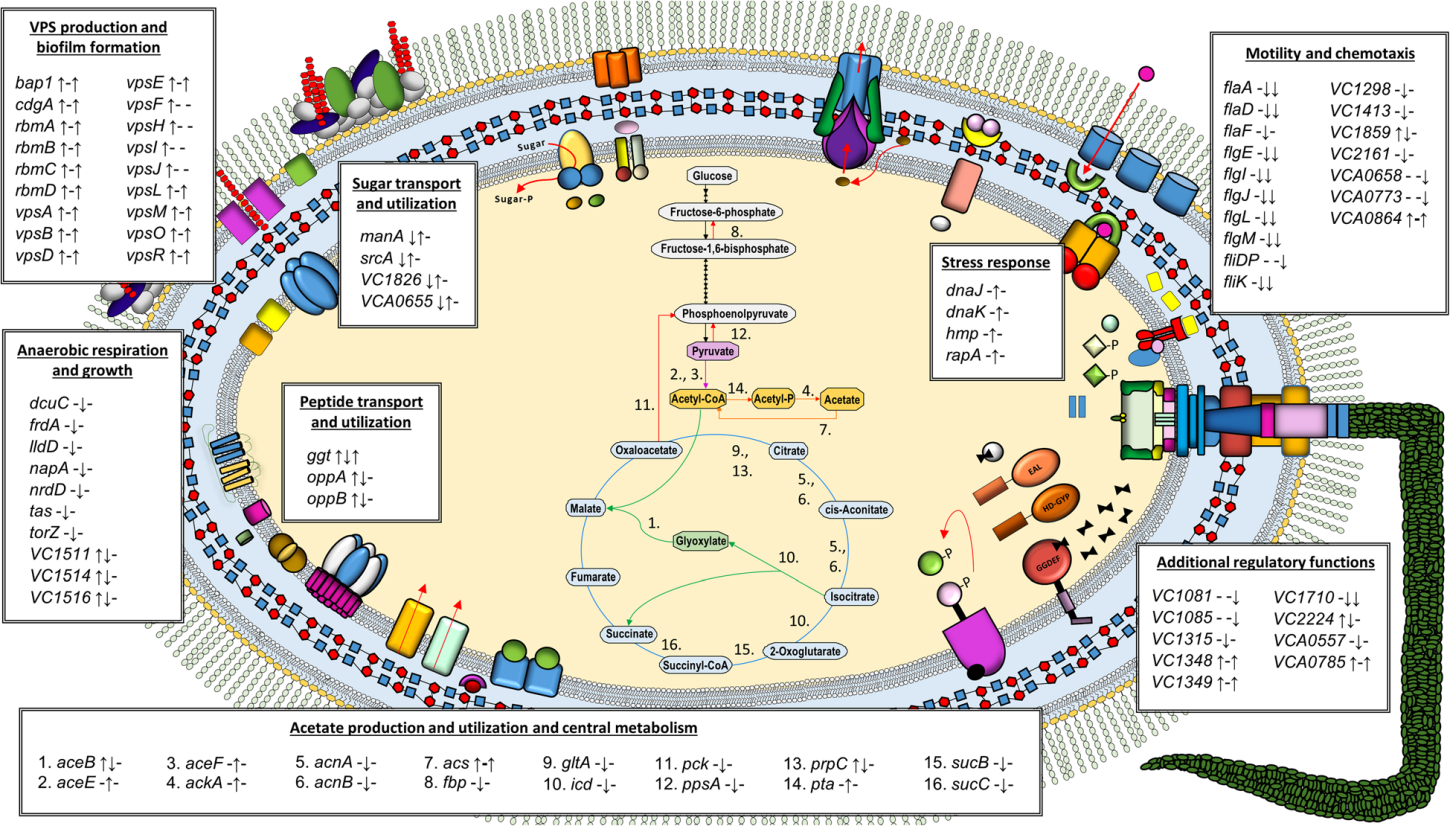
Overview of the differentially regulated genes discussed in this paper. The first symbol next to each gene name represents the qualitative expression change from N16961 to N16961R, the second symbol denotes the change from N16961R to N16961SD and the third represents the difference from N16961 to N16961SD. Symbol types are as follows: a dash indicates no significant change in gene expression, an upward arrow indicates up-regulation of the gene, and a downward arrow indicates down-regulation of the gene. Summary of central metabolic pathways shown within the cell includes the locations of gene functions as labeled with numbers. Pathways or portions of a pathway are colored as follows: red = gluconeogenesis; purple = pyruvate dehydrogenase complex; gold = acetate metabolism; green = glyoxylate bypass; blue = TCA cycle. As identified in this study, genes with the expression pattern ↑-↑ (or ↑↓↑ in the case of *ggt*) compose the transcriptomic signatures shared between N16961R and N16961SD strains

A summary of the differentially regulated genes discussed here is presented in Fig. 6. Besides the *vps*/*rbm* signature, other expression pathways that were up-regulated in N16961R and N16961SD relative to N16961 included a putative two-component regulatory system (VC1348, VC1349), c-di-GMP synthesis (VCA0785) and chemotaxis (VCA0864), as well as the *ggt* gene, which has been implicated in the environmental persistence of several different bacterial species [41–44]. Taken together, our results suggest these transcriptomic signatures may represent a stress adaptive consequence which allows for a more rapid phenotypic response (e.g., a switch from smooth back to rugose) when potentially changing environmental conditions dictate. Additional characterization of N16961SD should further our understanding of the role of this variant in the ecology and pathogenesis of strain N16961. It would also be interesting to determine whether this phenotype arises in populations of other pandemic and non-pandemic *V. cholerae* strains. Lastly, the differentially regulated genes identified here have provided additional insights into the multitude of underlying changes that occur with phase variation in this bacterial pathogen.

## Methods

### Bacterial strains and growth conditions

The *V. cholerae* phase variants used were smooth and rugose derivatives of O1 El Tor strain N16961 obtained from ATCC (Manassas, VA). All phase variants were isolated by daily passaging and occasional plating (Fig. 1) in Luria-Bertani (LB) broth (Difco, BD Diagnostics, Sparks, MD) supplemented to 2% NaCl (Fisher, Hampton, NH) at 30 °C with shaking at 200 rpm. Phase variants were stored as frozen stocks in LB broth with 2% NaCl and supplemented with 15% glycerol (Mallinckrodt, St. Louis, MO). All subsequent work, including RNA isolation, quantitative switching assays and further phenotypic characterization, including pellicle production and biofilm formation, was performed in LB broth containing 1% NaCl. It is noteworthy that for a given isolate there was no discernible change in colonial phenotype on LB medium containing 2% versus 1% NaCl.

### Quantitative switching assays

Quantitative switching assays to determine frequencies of phase variation were performed as previously described [47] with the following modifications. For each phase variant, three isolated colonies were selected and each was inoculated into 3 ml of fresh broth media. Each culture was incubated with shaking overnight, and then diluted 1:100 into three tubes of 3 ml fresh media. The tubes were passaged daily by diluting 1:100 into 3 ml of fresh media. After every fifth passage, serial dilutions of each culture were plated onto LB plates. Colony phenotypes were then scored and switching frequencies were calculated as described [48].

### Biofilm assays

Biofilm assays were performed as previously described [2, 48] with several modifications. Overnight cultures were diluted 1:100 into 1 ml of media in borosilicate glass tubes and incubated statically at 30 °C for 48 h. Cultures were then poured off and pellicles were photographed using a Sony Cybershot digital camera (Japan). The tubes were then rinsed with a 1.5% NaCl solution to remove pellicles and all non-attached cells. The remaining biofilms were stained with 0.1% crystal violet (CV) (Sigma-Aldrich, Saint Louis, MO), incubated for 30 min at room temperature, and rinsed again three times with 1.5% NaCl solution to remove excess CV. The remaining biofilm-attached CV was solubilized with 1.1 ml of DMSO (Sigma-Aldrich) and quantified by measuring the absorbance at 570 nm. Six independent replicates of each phase variant plus three uninoculated control replicates were used per assay for a total of two trials. Significance was determined using one-way ANOVA and Tukey’s post-test with a cutoff value of *P* ≤ 0.05.

### Motility assays

A total of ten plates containing 0.3% agar were inoculated with isolated colonies of each phase variant using an inoculating needle as previously described [47]. The plates were incubated at 30 °C overnight above a container of water to prevent dehydration of the motility agar. Following overnight incubation, each motility zone was measured in mm, and the plates were photographed with a Gel Doc XR (Bio-Rad Laboratories, Hercules, CA) using the EPI white setting and phase variant names were labeled on photographs using Microsoft Paint. Significance was determined using one-way ANOVA and Tukey’s post-test with a cutoff value of *P* ≤ 0.05.

### Growth curves

Growth curves of phase variant cultures were performed as previously described [48] except that time points were taken every 30 min until 5 h of incubation.

### RNA isolation

Freezer stocks were used to inoculate a 3 ml broth culture for each phase variant. Cultures were incubated overnight and streaked for isolation. Isolated colonies of each phase variant were inoculated into 3 ml broth cultures. Following overnight incubation, each culture was diluted 1: 200 into fresh media for RNA isolation and incubated with shaking until an OD_600_ of approximately 0.4, corresponding to mid-exponential growth phase, was reached. In order to achieve more synchronized growth, the cultures at that point were diluted at 1:100 into fresh media and incubated with shaking until an OD_600_ of approximately 0.4 was again reached. Multiple 1 ml aliquots were harvested and total RNA was isolated as previously described [49]. Each culture used for RNA isolation was streaked onto LB agar and observed for evidence of switching following overnight incubation. If observable switching occurred, the corresponding RNA samples were discarded and RNA isolation was repeated for that sample. The concentration and quality of each RNA sample was determined using the A_260_/A_280_ values reported with a Nanodrop spectrophotometer (Thermo Scientific, Wilmington, DE), and the integrity of the total RNA was further determined using agarose gel electrophoresis.

### Library preparation and sequencing

Total RNA was sent to the University of Illinois at Urbana-Champaign Roy J. Carver Biotechnology Center for library preparation and sequencing. Total RNA was depleted of ribosomal RNA using the RiboZero Metabacteria kit (Epicentre, Madison, WI) following the manufacturer’s instructions. The rRNA-depleted samples were then chemically fragmented to sizes ranging from 400 to 500 nucleotides. To create 5’ to 3’ strand-specific cDNA libraries, a TruSeq Stranded Sample Preparation kit (Illumina, San Diego, CA) was used followed by gel extraction to select fragments with a minimum size of 80 nucleotides. Fragments were barcoded and multiplexed on a single lane of an Illumina HiSeq2000 following the manufacturer’s instructions for 101 cycles. From an RNA population ranging in sizes from 80 to 500 nucleotides, 100 nucleotides were sequenced from randomly selected ends using the TruSeq SBS Sequencing Kit Version 3 and demultiplexed with Casava 1.8.2 following the manufacturer’s instructions.

### Data analysis

Sequences were checked for low quality reads and enrichment of artifacts such as adapters using FASTQC (http://www.bioinformatics.babraham.ac.uk/projects/fastqc/). All samples were of very high quality (Phred quality scores > 30), so further processing was not required for downstream analyses. End-to-end read alignment to the reference genome (NCBI GenBank accession numbers AE004093-AE004343, AE004344-AE004436) was performed using BowTie2 version 2.0.0-beta5 [50] with the “very sensitive” alignment preset to obtain an overall alignment rate for all samples of > 99%. Reads that did not entirely map well within the boundaries of individual predicted ORFs were filtered out from further analysis. The remaining uniquely mapped reads from the three biological replicates of each phase were pooled together to make groups N16961, N16961R, and N16961SD. These groups were analyzed in three pairwise comparisons (i.e., N16961 vs. N16961R, N16961R vs. N16961SD, and N16961 vs. N16961SD) using the statistics program R version 3.0.1 (R Core Team) with the package “DESeq”, Bioconductor version 2.14 [29] to determine differential expression based on the negative binomial distribution model, which is useful when applied to datasets with an unbounded positive range in which the sample variance may exceed the sample mean. Included in the DESeq results tables (Additional file 7: Table S3; Additional file 8: Table S4; Additional file 9: Table S5) are base means, which were calculated as the mean of all read counts for each gene, normalized to the total library size for that pairwise comparison and averaged over all 6 samples for that comparison. The base mean values for individual groups are the mean read counts from all 3 samples of that group still normalized by total library size per comparison. Fold changes and the logarithm of fold changes (to basis 2) from the first group to the second group are reported in the tables, along with the values for statistical significance (*P*). False discovery rate was controlled at 5% using the Benjamini and Hochberg method [30].

### RT-qPCR (Reverse Transcription Quantitative Real-Time PCR)

Primers were designed using Clone Manager (Sci-Ed Software; Morrisville, NC), and were verified in silico with Primer-BLAST (NCBI) prior to being synthesized by Sigma-Genosys (The Woodlands, TX). Primer sequences were as follows: *vpsA*-F 5’-TACCACGTTTGCTGCCTCTT-3’, *vpsA*-R 5’-AACCCGCTTCAACATGACCT-3’, *vpsL*-F 5’-CGCTTGGTTTGTCGGTTCTT-3’, *vpsL*-R 5’-AGTGAATGGTCGCAAATGCC-3’, *rbmC*-F 5’-GTATCAAGCGAACGATGCGG-3’, *rbmC*-R 5’-AAAGTGGCAGGTACAGAGGC-3’. Primers used for the reference gene, *gyrA*, were those previously published [51]. Primers were verified in vitro by standard PCR and subsequent agarose gel electrophoresis using *V. cholerae* N16961 genomic DNA (gDNA), which was purified as described previously [49]. For cDNA, first strand synthesis was performed as described [49]. Primer efficiencies to determine appropriate primer and cDNA concentrations were conducted in duplicate using five serial 1:10 dilutions of *V. cholerae* N16961 cDNA for each gene target and reference gene and with the following controls: water + reverse transcriptase (RT), gDNA -RT, and non-template controls (NTC), run on a ViiA7 Real Time PCR System (Applied Biosystems, Carlsbad, CA) using SYBR Select Master Mix chemistry (Applied Biosystems). Numerical efficiency was determined by the formula E = 10^(‐ 1/slope)^ ‐ 1, and all calculations were made within the Expressionsuite software v1.0.3 (Applied Biosystems). RT-qPCR was conducted on each sample versus each gene target in triplicate with 0.5 μl of appropriate 20 mM forward and reverse primers, 5 μl of cDNA diluted 1:100 in nuclease free (NF) water, 12.5 μl SYBR Select Master Mix, and water to 25 μl. Samples were run alongside NTC, gDNA -RT, and water + RT controls on 96-well plates in the ViiA7 Real Time PCR System, and gene expression was determined using the relative standard curve method for relative quantification within the accompanying Expressionsuite software v1.0.3. Each assay was repeated three times.

### Figure preparation

Additional file 4: Figure S2; Additional file 5: Figure S3; Additional file 6: Figure S4 were created using R software with the “gplots” package version 2.12.1 [52]. Fig. 4 was plotted using R software with the package “FactoMineR” version 1.25 [53]. In Fig. 5a, peaks corresponding to mapped RNA transcripts were visualized in parallel tracks against the *V. cholerae* N16961 genome using Integrative Genomics Viewer [35]. These tracks were scaled to normalize for differences in the total number of read counts of each sample. Whole colony images for Fig. 1 were taken using a Zeiss SteREO Lumar.V12 microscope with the brightfield setting.

## Additional files

Additional file 1: Figure S1. Growth curves of N16961 phase variants. Plotted are the means of 9 replicates of N16961 and 3 of each of the individual N16961R and N16961SD phase variants used in this study (i.e., *n* = 9 [total] for N16961R and 9 [total] for N16961SD) on a semi-logarithmic (base 10) graph. Error bars indicate standard deviation. (TIF 1479 kb)
Additional file 2: Table S1. Percentage of reads mapped to the reference genome for each RNA sample. (XLSX 10 kb)
Additional file 3: Table S2. Total fragments mapped exclusively within each predicted gene per sample and normalized FPKM values. (XLSX 471 kb)
Additional file 4: Figure S2. Heatmap showing global gene expression in N16961 versus N16961R comparison. Relative gene expression values for each phase variant are represented by variations in color as depicted in the color key. (TIF 481 kb)
Additional file 5: Figure S3. Heatmap showing global gene expression in N16961R versus N16961SD comparison. Relative gene expression values for each phase variant are represented by variations in color as depicted in the color key. (TIF 472 kb)
Additional file 6: Figure S4. Heatmap showing global gene expression in N16961 versus N16961SD comparison. Relative gene expression values for each phase variant are represented by variations in color as depicted in the color key. (TIF 467 kb)
Additional file 7: Table S3. Results of DESeq analysis for the N16961 to N16961R pairwise comparison. (XLSX 190 kb)
Additional file 8: Table S4. Results of DESeq analysis for the N16961R to N16961SD pairwise comparison. (XLSX 192 kb)
Additional file 9: Table S5. Results of DESeq analysis for the N16961 to N16961SD pairwise comparison. (XLSX 183 kb)
Additional file 10: Table S6. Genes that were significantly up-regulated from N16961 to N16961R and remained up-regulated in N16961SD. (XLSX 14 kb)
Additional file 11: Table S7. Genes that were significantly up-regulated from N16961 to N16961R and were significantly down-regulated from N16961R to N16961SD. (XLSX 13 kb)
Additional file 12: Table S8. Genes that were significantly down-regulated from N16961 to N16961R and were significantly up-regulated from N16961R to N16961SD. (XLSX 10 kb)
Additional file 13: Table S9. Genes that were not significantly differentially regulated from N16961 to N16961R but were significantly down-regulated in N16961SD. (XLSX 25 kb)
Additional file 14: Table S10. Genes that were not significantly differentially regulated from N16961 to N16961R but were significantly up-regulated in N16961SD. (XLSX 22 kb)

## Abbreviations

ACKA: Acetate kinase
ACS: Acetyl-coenzyme A synthetase
DGC: Diguanylate cyclase
FPKM: Fragments per kilobase per million reads
GB: Glyoxylate bypass
GGT: Gamma-glutamyl transpeptidase
PCA: Principal component analysis
PDC: Phosphodiesterase
PTA: Phosphate acetyltransferase
PTS: Phosphoenolpyruvate phosphotransferase system
VPS: *Vibrio* polysaccharide
VPS-PS: Polysaccharide portion of *Vibrio* polysaccharide
