# Supplementary figures and images for: A novel phase variant of the cholera pathogen shows stress-adaptive cryptic transcriptomic signatures
Source: BMC Genomics. 2016 Nov 14;17:914. doi: 10.1186/s12864-016-3233-x (PMC5109742; doi:10.1186/s12864-016-3233-x)

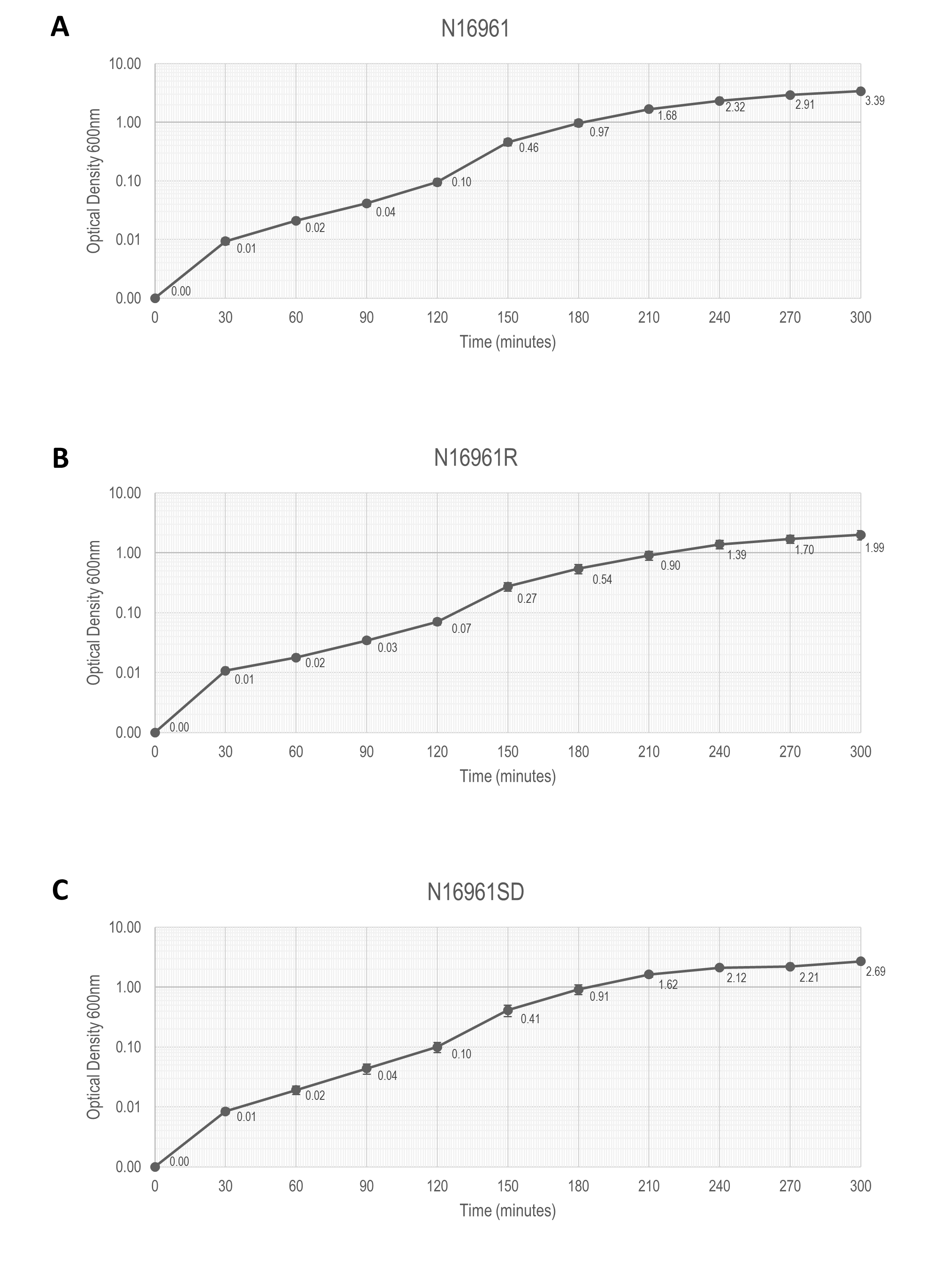

Supplement: Additional file 1: Figure S1. — Growth curves of N16961 phase variants. Plotted are the means of 9 replicates of N16961 and 3 of each of the individual N16961R and N16961SD phase variants used in this study (i.e., n = 9 [total] for N16961R and 9 [total] for N16961SD) on a semi-logarithmic (base 10) graph. Error bars indicate standard deviation. (TIF 1479 kb) [file 12864_2016_3233_MOESM1_ESM.tif]

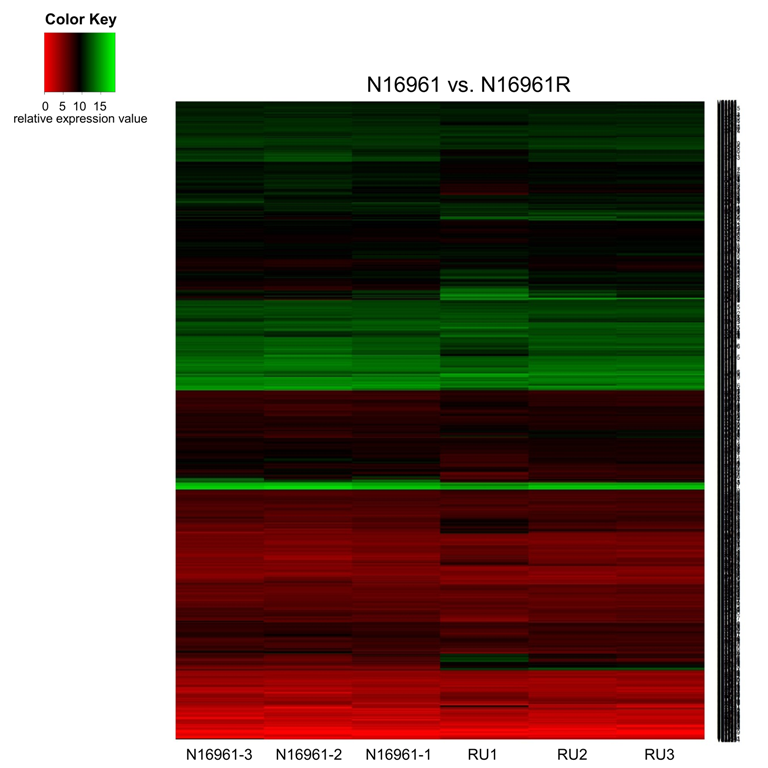

Supplement: Additional file 4: Figure S2. — Heatmap showing global gene expression in N16961 versus N16961R comparison. Relative gene expression values for each phase variant are represented by variations in color as depicted in the color key. (TIF 481 kb) [file 12864_2016_3233_MOESM4_ESM.tif]

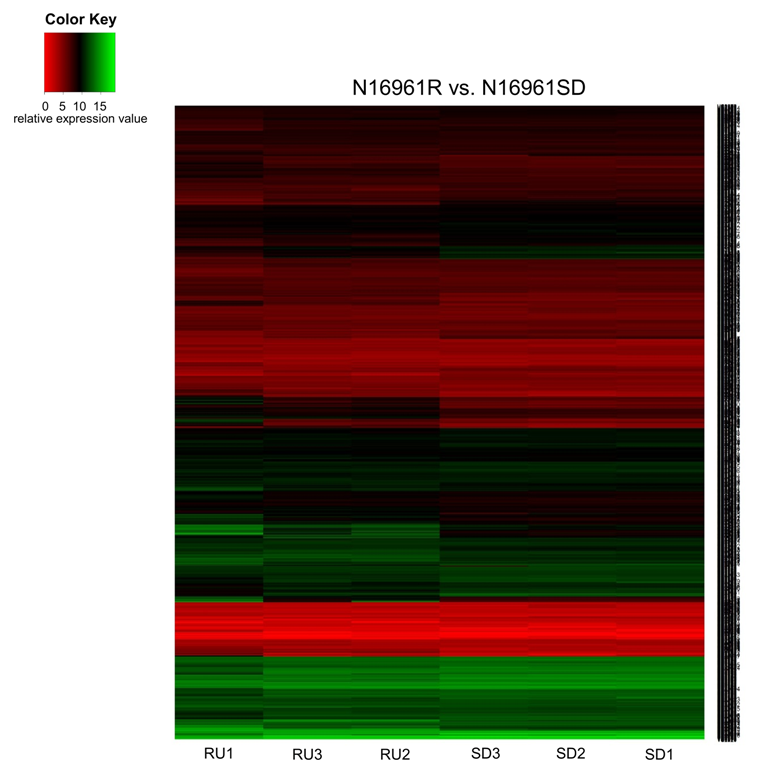

Supplement: Additional file 5: Figure S3. — Heatmap showing global gene expression in N16961R versus N16961SD comparison. Relative gene expression values for each phase variant are represented by variations in color as depicted in the color key. (TIF 472 kb) [file 12864_2016_3233_MOESM5_ESM.tif]

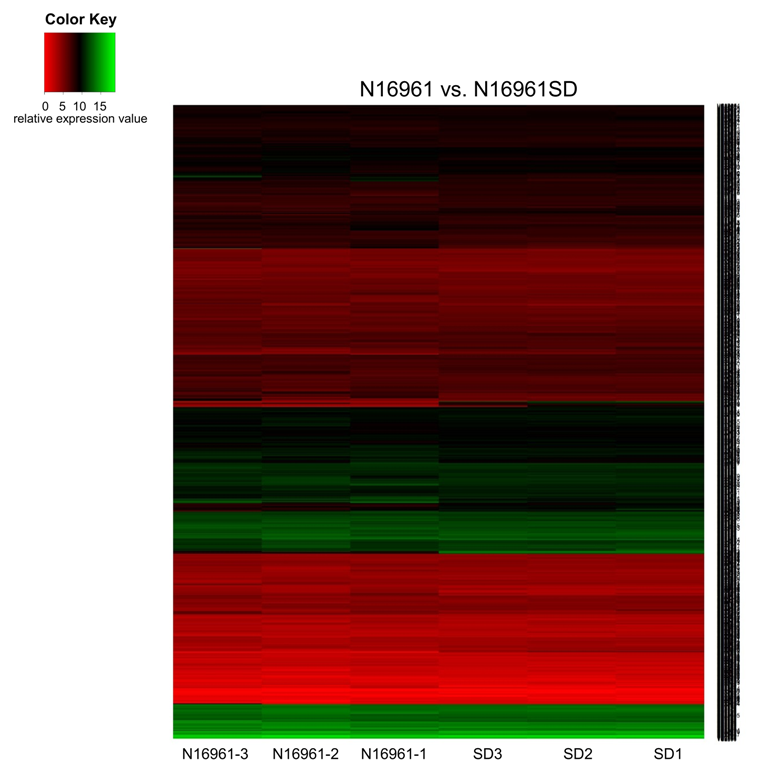

Supplement: Additional file 6: Figure S4. — Heatmap showing global gene expression in N16961 versus N16961SD comparison. Relative gene expression values for each phase variant are represented by variations in color as depicted in the color key. (TIF 467 kb) [file 12864_2016_3233_MOESM6_ESM.tif]
